# A single dose of oral nattokinase accelerates skin temperature recovery after cold water immersion: A double-blind, placebo-controlled crossover study

**DOI:** 10.1016/j.heliyon.2023.e17951

**Published:** 2023-07-06

**Authors:** Noriko Nara, Yuko Kurosawa, Sayuri Fuse-Hamaoka, Miyuki Kuroiwa, Tasuki Endo, Riki Tanaka, Ryotaro Kime, Takafumi Hamaoka

**Affiliations:** a Department of Sports Medicine for Health Promotion, Tokyo Medical University, 6-1-1 Shinjuku, Shinjuku-ku, Tokyo 160-8402, Japan; b Department of Food & Health Sciences, Jissen Women's University, 4-1-1 Osakaue, Hino-shi, Tokyo 191-8510, Japan; c Faculty of Science and Technology, Meijo University, 1-501 Shiogamaguchi, Tempaku, Nagoya, Aichi 468-8502, Japan

**Keywords:** Blood pressure, Cardiac output, Cold water immersion, Peripheral circulation, Sympathetic nervous activity, Visual analogue scale

## Abstract

Nattokinase (NK) intake may improve blood flow; however, its effects on skin temperature, which is predominantly controlled by skin surface blood flow, are unknown. The purpose of this study was to determine the effects of a single dose of NK on changes in skin temperature after cold water immersion. A double-blinded, placebo-controlled, crossover intervention study was performed on nine healthy men. The participants were randomised to receive either a single dose of 2,000 fibrinolytic units (FU) of NK or a placebo with subsequent crossover. Two hours after supplementation, the participants immersed both hands in a water bath maintained at 10 °C for 1 min. Skin temperature, perceived coldness, cardiac output, and sympathetic nervous activity were measured before, during, and after water immersion. Two-way analysis of variance showed a significant effect of treatment interaction on the skin temperature of the middle finger, palm, and back of the right hand (p < 0.05). These findings represented that the skin temperatures of the middle finger, palm, and back of the right hand immersed in the cold water were significantly dropped due to the cold water immersion, and then recovered more quickly by NK intake than by placebo intake. The results of the current study highlight the potential implications of NK for the prevention of excessive vasoconstriction. It may be more significant for those with cold-sensitive constitution, such as women and elderly. In contrast, the acute administration of 2,000 FU of NK did not affect changes in heart rate, cardiac output, sympathetic nervous activity compared with a placebo in healthy men.

## Highlights

• A single dose of NK intake enhances peripheral skin temperature after cold exposure.
• A single dose of NK intake does not affect resting blood pressure in healthy men.
• Acute NK intake may be applicable for the prevention of cold sensitivity.

## Graphical abstract

**Figure:**
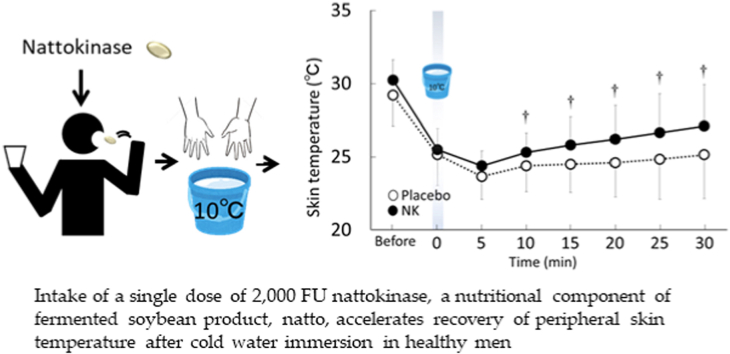

## 1 Introduction

One of the unique characteristics of traditional Japanese-style diets is that they contain abundance of soy-beans [1], and the interaction of fermented soybean products, such as natto and miso, with certain *Bacillus* species results in a variety of ingredients with functional nutrients [2]. The Takayama study, a community-based large cohort study in Japan, revealed that natto intake, but not soy protein or soy isoflavone intake, is significantly associated with a decreased risk of mortality from cardiovascular diseases [3]. Thus, fermented soybean products have received significant attention for their health benefits. Nattokinase (NK), a nutritional component of the fermented soybean product, “natto”, is a single polypeptide chain enzyme of 275 amino acids with a molecular weight of 27.7 kDa (Km, 7.43 × 10^−4^ M; Vmax, 0.088 μmol/min) [4]. NK possesses features that degrade blood clots directly and indirectly through several different pathways in vivo [[5], [6], [7], [8]]. Oral intake of NK may enhance immune function by activating natural killer cells in healthy humans [9]. Previous studies have also demonstrated that oral NK intake favoured haemodynamic responses by reducing blood pressure, in particular, decreasing peripheral vascular resistance, both in animals and humans [10,11].

Environmental exposure to cold challenges the homeostasis of the core body temperature, in which the peripheral skin vascular network plays an important role in shifting peripheral blood flow to the body's core [12]. Peripheral skin blood flow is important, not only for maintaining skin temperature but also for delivering oxygen and nutrients and retrieving waste products. Optimal regulation of vasoconstriction/vasodilation is critical for normal physiological function and prevention of pathological symptoms, such as pressure ulcers and ischaemic necrosis. The cold water immersion test, which is used to evaluate vasomotor nerve function in response to cold exposure, is a screening technique for peripheral circulatory disorders such as hand-arm vibration syndrome [13] and systemic sclerosis [14]. Watanabe et al. [15] reported that a single dose of oral NK in humans enhanced blood flow, a key regulator of skin temperature, in the middle finger of both hands; however, the effects of oral NK intake on changes in peripheral skin temperature and related parameters, such as haemodynamic response and sympathetic nervous activity, due to cold water immersion are yet to be investigated.

Here, we hypothesised that a single dose of oral NK would accelerate recovery of skin temperature after cold water immersion. This double-blinded, placebo-controlled crossover study, which aimed to examine the effects of acute NK intake on changes in skin temperature after cold water immersion, found that a single dose of NK enhanced recovery from cold-induced decrease in peripheral skin temperature in healthy Japanese men.

## 2 Materials and methods

### 2.1 Study participants

**Fig. 1:**
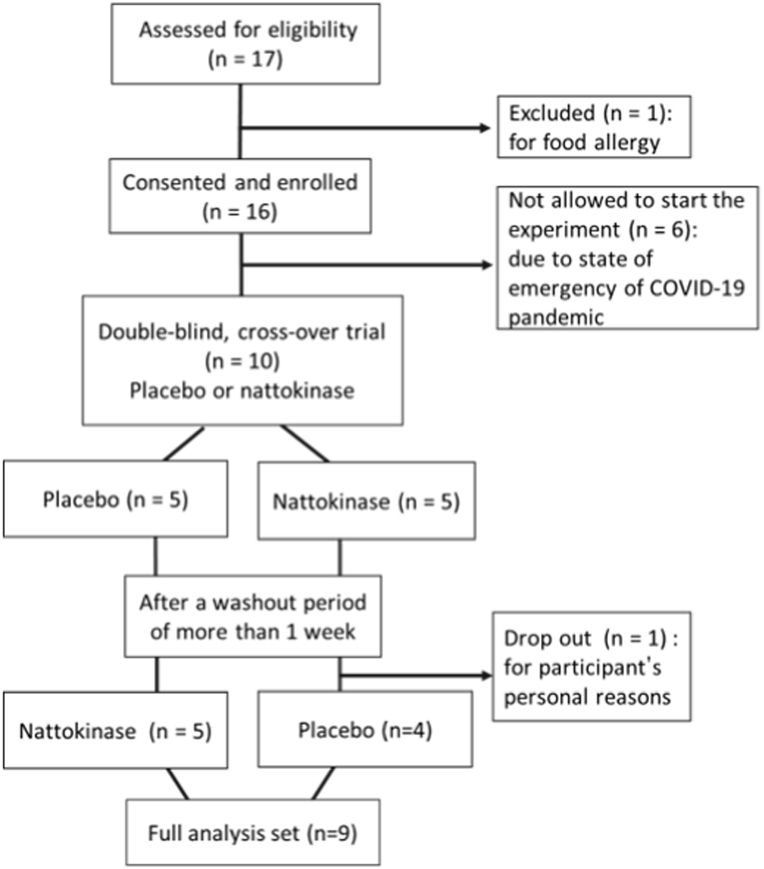
Flowchart of the participant selection process.

Nine healthy men participated in this study. Participants were recruited through advertisements on posters and the Internet, or through direct contact. Seventeen men who responded to the advertisements were invited for screening. After screening, we excluded one candidate with a food allergy. Additionally, six participants could not participate in the experiment due to the declaration of the state of emergency for the coronavirus disease pandemic. Furthermore, another candidate dropped out before the second experiment for personal reasons. Finally, nine healthy Japanese men participated in this study (Fig. 1). None of the participants were sensitive to cold when evaluated using a questionnaire [16]. The participants were normotensive non-smokers with no medication for or clinical history of haematologic disease or symptoms of venous and arterial diseases. All participants stated that they had never taken the NK supplement, and that they had not consumed food with natto within 2 weeks before the experiment started. The same researchers performed all the procedures using identical techniques. All experimental procedures and measurements conformed to the Declaration of Helsinki and were approved by the Institutional Review Board of Tokyo Medical University (#T2019-0047). This trial was registered with the University Hospital Medical Information Network (UMIN000045964). All participants were fully informed of all procedures and their potential risks and provided oral and written informed consent before participating in this study.

### 2.2 Experimental procedures

**Fig. 2:**
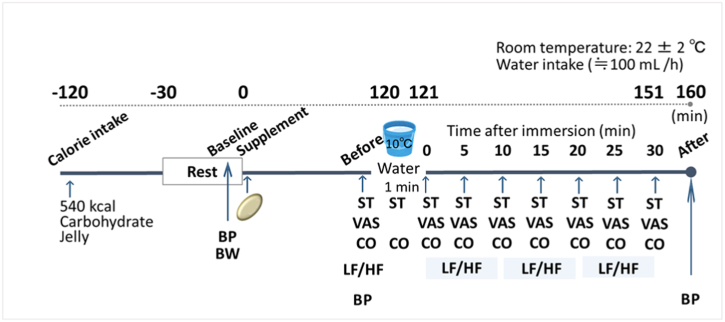
Schematic diagram of the experimental procedures. Blood pressure (BP) was measured at baseline (before supplementation), before (90 min after supplementation), and after (160 min after supplementation) cold water immersion of hands. Body weight (BW) was measured at baseline. Following baseline measurement, each participant was randomised to receive either a single dose of 2,000 fibrinolytic units (FU) NK or placebo. Skin temperature (ST), perceived coldness (visual analogue scale, VAS), cardiac output (CO) and related parameters, and low frequency/high frequency ratio (LF/HF) were measured before, during, and after cold water immersion.

A schematic representation of the study design illustrating the sequence of events is shown in Fig. 2. A double-blinded, placebo-controlled, crossover intervention study was performed on nine healthy men. The participants were randomly allocated to receive the placebo or NK supplement by a non-participating third party at each trial. In addition to the participants, all investigators and data analysts were blinded until the data were analysed. Participants were instructed to refrain from any form of exercise, avoid intake of high-fat and high-protein diets, alcohol, and caffeine 24 h before the experiment, and to consume dinner by 8 p.m. on the day prior to the experiment. Participants were instructed to arrive at the laboratory 90 min after consuming 540 kcal of a jelly-type carbohydrate drink containing 402 mL of water stored at room temperature, to minimize diet-induced thermogenesis. Each participant arrived at the laboratory between 7:30 a.m. and 8:30 a.m. to minimize potential diurnal variations. All experiments were performed in a temperature-controlled room that was maintained at 22 ± 2 °C. The participants were allowed a 30-min acclimatisation period in the temperature-controlled room wearing unified clothes (long sleeve shirt and long pants). Following baseline measurements of blood pressure and body weight, each participant was randomised to receive either a single dose of 2,000 fibrinolytic units (FU) of NK (containing 3.97 mg of NK) in the form of a soft gel capsule (110 mg of NSK-SD [the product name of nattokinase supplement], Japan Bio Science Laboratory Co., Ltd, Osaka, Japan) or a soft gel capsule containing the placebo. A dose of 2,000 FU of NK has already been validated with regard to bioavailability [17], safety [18], and efficacy for the improvement of peripheral blood flow in humans [15]. After a washout period of 14 days on average, the second trial commenced with an alternate intake.

Blood pressure, skin temperature, perceived coldness, cardiac output (CO) and related parameters, and low frequency to high frequency ratio (LF/HF) were measured before cold water immersion. Two hours after supplementation, both hands were immersed in a 10°C-water bath up to the radial styloid processes for 1 min. After 1 min, the participants removed their hands from the water bath, and their hands were wiped with towels to remove water drops. Both forearms were then rested on an upper limb stand for 30 min, while ensuring both hands were not in contact with the stand. Skin temperature, perceived coldness, and CO and related parameters were measured every 5 min, and the LF/HF ratio was monitored every 10 min after immersion for 30 min. Blood pressure was also measured 39 min after immersion. The participants were not permitted to eat but drank the same amount of water during the experiment (placebo: 108. 3 ± 14.9 mL/h, NK: 106.4 ± 14.0 mL/h). The participants went to the lavatory ad libitum during the study period. All nine participants completed the experiment, and no adverse effects were observed.

### 2.3 Skin temperature

**Fig. 3:**
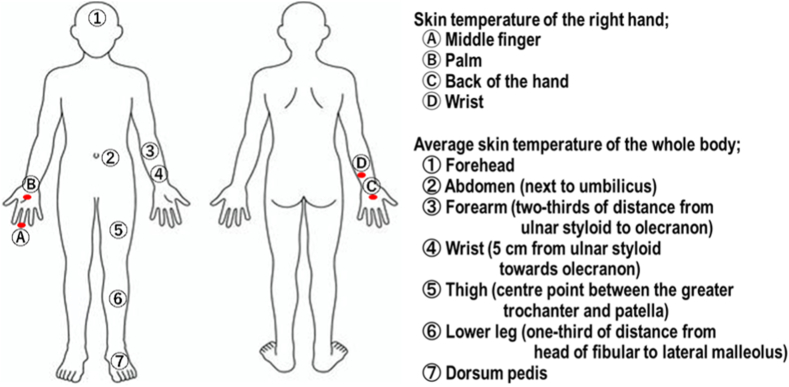
Attachment positions of the data loggers for skin temperature measurements; Ⓐ–Ⓓ for skin temperature of right hands, ①–⑦ for the average skin temperature of the whole body.

Skin temperature was monitored using a small disc-type temperature data logger (Thermochromics S, KN Laboratories, Osaka, Japan), which was placed at 11 positions during the experiments (Fig. 3).

The skin temperature of the middle finger, palm, wrist, and back of the right hand was monitored before, during, and every 5 min after immersion for 30 min to assess the effects of cold water immersion (Ⓐ-Ⓓ in Fig. 3). The data loggers of these four positions were covered with waterproof silicon. Using Hardy and BuBois’ formula [19], the mean skin temperature of the whole body was calculated using the skin temperatures of the forehead, abdomen, forearm, wrist, thigh, lower leg, and dorsum pedis (①-⑦ in Fig. 3) before, during, and every 5 min after water immersion as follows:

Mean skin temperature = 0.07 × ①＋0.14 × ③＋0.05 × ④＋0.35 × ②＋0.19 × ⑤＋0.13 × ⑥＋0.07 × ⑦

⁃ The data loggers of these seven positions, except ① (forehead) and ⑦ (dorsum pedis), were covered by the unified clothes during the experiments.

### 2.4 Perceived coldness

Perceived coldness in the hands before and after the cold water immersion was assessed using the visual analogue scale (VAS). The VAS used in this study was a self-report scale represented on a horizontal line of 156 mm and anchored by the ratings “no cold feeling” at the left side (score 0%) and “worst cold feel-ing” at the right side (score 100%). Perceived coldness was evaluated before; immediately; and 5, 10, 15, 20, 25, and 30 min after the cold water immersion.

### 2.5 CO and related parameters

Heart rate (HR), stroke volume, CO, left ventricular end-diastolic volume, and ejection fraction were monitored using a transthoracic electrical bioimpedance device (PhysioFlow®, Manatec type PF05L1, Paris, France) [20]. After gently scraping the skin, six electrodes (Echorode III, Fukuda Denshi, Inc., Tokyo, Japan) were placed on the skin at the following positions: two on the neck, two just under the xiphisternum, and one each on V1 and V5 on the chest as recommended in the manufacturer's instruction manual. After initial calibration, haemodynamic measurements were taken before, during, and every 5 min after cold water immersion for 30 min.

### 2.6 Sympathetic nervous activity

The LF/HF ratio, as an indicator of sympathetic nervous activity, was compared before and after cold water immersion. First, the variability of the fingertip pulse wave of the left index finger was obtained by power spectral analysis (TAS9, YKC Co., Tokyo, Japan). Then, the LF and HF components were calculated using a HR variability software program (HRV analysis ver. 1.2., University Saint Etienne, France) [21] using the fast Fourier transformation: LF (0.04–0.15 Hz) and HF (0.15–0.4 Hz). The LF/HF ratio was calculated before and every 10 min after the cold water immersion.

### 2.7 Blood pressure

Systolic and diastolic blood pressures of the left hand were measured in the sitting position before supplementation (baseline), 90 min after supplementation (30 min before immersion), and 160 min after supplementation (39 min after completion of water immersion) once each time using a digital automatic blood pressure monitor (HEM-1021, OMRON, Co., Ltd, Kyoto, Japan).

### 2.8 Body weight

Body weight was assessed at baseline using multifrequency bioelectrical impedance measurements with eight tactile electrodes (InBody 720; Biospace, Tokyo, Japan).

### 2.9 Sample size and statistics

#### 2.9.1 Sample size

Our primary outcome was skin temperature during and after cold water immersion of the hands; however, no previous studies have evaluated the effects of acute NK intake on skin temperature. A study conducted by Watanabe et al. (2018) [15]. Reported that a single dose of 2,000 FU of NK enhanced skin blood flow in the right and left middle fingers in healthy participants (placebo; before: 2.11 ± 0.05 V, after: 2.21 ± 0.12 V, NK; before: 1.89 ± 0.09 V, after: 2.31 ± 0.06 V [mean ± standard deviation (SD)]). Thus, we calculated the sample size for this study, using G power software (version 3.1, Bonn University, Bonn, Germany), based on this previous work [15]. Using an 80% power and 95% confidence level, nine participants were required to detect a significant difference between NK and placebo intakes (critical t = 1.859, p < 0.05).

#### 2.9.2 Statistical analysis

The effects of NK intake on skin temperature, perceived coldness, CO and related parameters, sympathetic nervous activity, and blood pressure were analysed based on treatment (placebo vs. NK) and time (before, during, and after completion of the cold water immersion) using the repeated-measures two-way analysis of variance (ANOVA), followed by Bonferroni's multiple comparison tests, if necessary. Quantitative results are presented as mean ± SD. Statistical analyses were performed using the Japanese SPSS version 27 (IBM SPSS Japan, Tokyo, Japan), and the level of statistical significance was set at < 5% for all tests.

## 3 Results

Nine participants (age, 29.8 ± 15.2 years; height, 169.0 ± 5.1 cm; body weight, 62.5 ± 5.9 kg; body mass index [BMI], 21.9 ± 2.3 kg/m^2^; %body fat, 17.9 ± 4.7%) completed the study. No side effects of the supplements or cold water immersion were observed.

### 3.1 Skin temperature

**Fig. 4:**
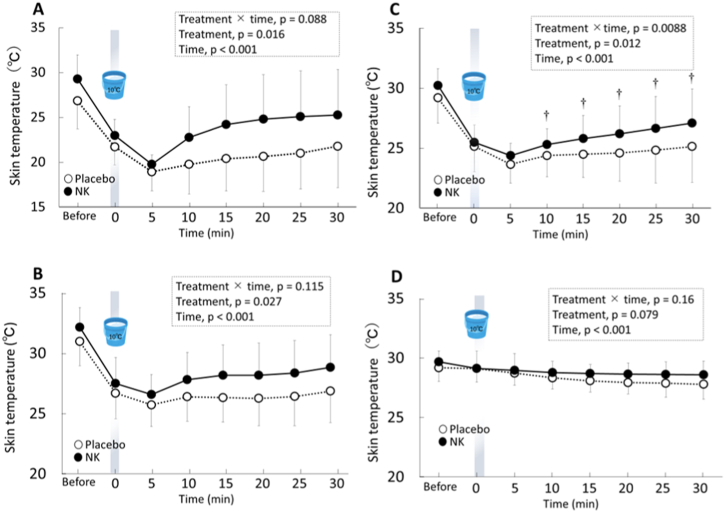
Changes in skin temperature of the middle finger (A), palm (B), back (C), and wrist (D) of the right hands after placebo (open symbols) and NK (closed symbols) intake before, during, and after cold water immersion of both hands at 10 °C for 1 min. Data are expressed as mean ± SD. P values for treatment (placebo vs. NK) and time (before, during, and after cold water immersion) were obtained using the repeated two-way analysis of variance, followed by Bonferroni's multiple comparison tests, if necessary. †p < 0.05, statistically significant difference between placebo and NK in each time point analysed by Bonferroni's multiple comparison tests.

Skin temperature measurements before; during; and 5, 10, 15, 20, 25, and 30 min after cold water immersion of the right hand are shown in Fig. 4A–D. Repeated-measures ANOVA showed that interaction (treatment × time) and simple main effects of treatment and time had a significant effect on changes in skin temperature of the back of the right hand (Fig. 4C, p < 0.05), while the main effects of treatment and time had a significant effect on changes in skin temperature of the middle finger and palm of the right hand due to the cold water immersion (Fig. 4A and B, p < 0.05). This indicated that the skin temperature of the middle finger, palm, and back of the right hand decreased significantly due to the cold water immersion and, then, recovered more quickly after NK intake than placebo intake. By contrast, the skin temperature of the right wrist (Fig. 4D), which was not immersed in the cold water, and the mean skin temperature of the whole body (Table 1) changed significantly over time, but no differences were found between placebo and NK intakes. Furthermore, there was no significant difference in forehead skin temperature, as an indicator of core temperature, over time or between treatments (Table 1).

**Table 1:** Changes in skin temperature of whole body (mean) and forehead after placebo and NK intake before, during, and after cold water immersion of the hands.

		Before	Water	After (min)						*P*-value		
				5	10	15	20	25	30	Interaction	Treatment	Time
Whole body (°C)	P	32.0 ± 0.4	31.9 ± 0.4	31.9 ± 0.3	31.9 ± 0.3	31.8 ± 0.3	31.8 ± 0.3	31.7 ± 0.1	31.7 ± 0.0	0.26	0.07	<0.05
	NK	32.4 ± 0.6	32.4 ± 0.6	32.3 ± 0.6	32.3 ± 0.6	32.3 ± 0.5	32.3 ± 0.6	32.4 ± 0.6	32.3 ± 0.5			
Forehead (°C)	P	34.1 ± 1.0	34.1 ± 1.0	34.1 ± 1.1	34.1 ± 1.1	34.0 ± 1.1	34.0 ± 1.1	34.0 ± 1.1	34.2 ± 1.1	0.31	0.64	0.64
	NK	34.2 ± 0.9	34.2 ± 0.9	34.2 ± 1.0	34.3 ± 0.8	34.2 ± 0.8	34.2 ± 0.9	34.2 ± 0.9	34.1 ± 1.1			

Data are expressed as mean ± standard deviation. The p-values for interaction, treatment (placebo vs. NK), and time (before, during, and after cold water immersion) were obtained using the repeated-measures two-way analysis of variance, followed by Bonferroni's multiple comparison tests, if necessary. P; placebo, NK; nattokinase.

### 3.2 Perceived cold feeling

**Fig. 5:**
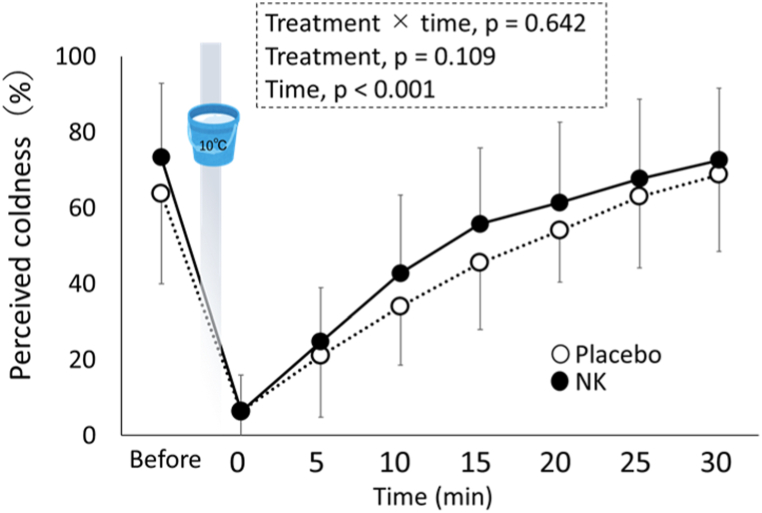
Changes in perceived coldness measured using the visual analogue scale for placebo (open symbols) and NK (closed symbols) before and after cold water immersion of both hands at 10 °C of water temperature for 1 min. Data are expressed as means ± SD. The p-values for treatment (placebo vs NK) and time (before and after cold water immersion), were obtained using the repeated two-way analysis of variance, followed by Bonferroni's multiple comparison tests, if necessary.

We found statistically significant differences in perceived coldness, measured using the VAS, over time, i.e., before; immediately; and 5, 10, 15, 20, 25, and 30 min after the cold water immersion of both hands; however, no differences were found between placebo and NK intakes (Fig. 5).

### 3.3 Cardiac output (CO) and related parameters

CO and related parameters, monitored before; during; and 5, 10, 15, 20, 25, and 30 min after cold water immersion of both hands, are shown in Table 2.

**Table 2:** CO and related parameters and LF/HF ratio after placebo and NK intake before, during, and after cold water immersion.

		Before	Water	After (min)						p-value		
				5	10	15	20	25	30	Interaction	Treatment	Time
HR (bpm)	P	64 ± 10	70 ± 7	62 ± 7	64 ± 10	64 ± 8	64 ± 10	67 ± 11	65 ± 8	0.27	0.76	<0.05
	NK	62 ± 10	73 ± 8	64 ± 11	62 ± 9	61 ± 7	64 ± 10	64 ± 7	63 ± 11			
SV (mL)	P	73.7 ± 13.0	66.5 ± 4.7	73.5 ± 15.4	73.5 ± 11.6	72.8 ± 13.6	73.4 ± 12.2	71.9 ± 10.9	72.2 ± 13.3	0.91	0.30	0.28
	NK	78.5 ± 16.0	70.7 ± 13.0	75.9 ± 15.7	75.8 ± 15.7	75.5 ± 14.8	76.7 ± 15.7	74.5 ± 13.3	77.8 ± 18.1			
CO (L/min)	P	4.7 ± 0.7	4.6 ± 1.0	4.5 ± 0.8	4.7 ± 0.7	4.6 ± 0.8	4.7 ± 0.8	4.8 ± 1.1	4.6 ± 0.7	0.25	0.35	0.54
	NK	4.8 ± 0.5	5.2 ± 1.0	4.7 ± 0.5	4.6 ± 0.6	4.5 ± 0.6	4.8 ± 0.5	4.7 ± 0.8	4.8 ± 0.8			
EDV (ｍL)	P	109.7 ± 20.4	103.0 ± 22.6	108.7 ± 21.6	109.9 ± 17.1	108.7 ± 20.4	109.6 ± 19.0	107.5 ± 15.7	107.3 ± 19.8	0.96	0.92	0.25
	NK	111.5 ± 18.5	104.2 ± 17.4	108.9 ± 20.3	109.2 ± 20.2	108.4 ± 20.4	109.5 ± 21.2	107.5 ± 18.7	109.9 ± 21.4			
EF (％)	P	67.5 ± 6.4	64.7 ± 5.2	67.7 ± 6.6	67.2 ± 6.8	67.2 ± 7.2	67.3 ± 7.0	67.2 ± 7.2	67.3 ± 6.4	0.87	0.20	0.23
	NK	70.3 ± 5.2	67.8 ± 4.7	69.5 ± 4.4	69.2 ± 4.8	69.6 ± 3.9	69.9 ± 4.4	69.3 ± 3.7	70.3 ± 3.9			
LF/HF ratio	P	1.1 ± 0.7			1.8 ± 1.0		2.0 ± 1.4		2.1 ± 1.5	0.04	0.89	<0.05
	NK	1.7 ± 1.0			1.1 ± 0.6		1.8 ± 1.0		2.2 ± 1.3			

Data are expressed as mean ± SD. The p-values for treatment (placebo vs. NK) and time (before, during, and after cold water immersion) were obtained using the repeated two-way analysis of variance, followed by Bonferroni's multiple comparison tests, if necessary. HR, heart rate; bpm, beats per minute; SV, stroke volume; EDV, left ventricular end-diastolic volume; EF, ejection fraction.

HR was significantly increased due to cold water immersion, whereas no difference in HR changes was found between the placebo and NK intakes (Table 2). No treatment interaction or differences in time were observed in all parameters, except for HR (Table 2).

### 3.4 Sympathetic nervous system activity

Changes in the LF/HF ratio, as an indicator of sympathetic nervous system activity, monitored before and 10, 20, and 30 min after cold water immersion are shown in Table 2. There was significant elevation in the LF/HF ratio due to the cold water immersion, but no differences were found between the placebo and NK intakes (Table 2).

### 3.5 Blood pressure

No significant differences were observed in the resting systolic and diastolic blood pressures before, 90 min, and 160 min after supplementation with NK or placebo (Table 3). A single dose of 2,000 FU of NK did not affect the resting blood pressure in this study.

**Table 3:** Systolic and diastolic blood pressure before and after placebo and NK intake at baseline, before, and after water immersion in nine healthy men.

		Baseline	Before water immersion	After water immersion	p-value		
					Interaction	Treatment	Time
Systolic blood pressure (mmHg)	p	111.0 ± 9.3	108.0 ± 10.3	107.0 ± 14.1	0.53	0.43	0.68
	NK	111.0 ± 10.6	110.0 ± 7.4	111.0 ± 15.9			
Diastolic blood pressure (mmHg)	p	71.0 ± 4.5	67.0 ± 8.9	68.0 ± 7.8	0.30	0.69	0.49
	NK	68.0 ± 6.1	68.0 ± 9.4	70.0 ± 9.7			

Data are expressed as mean ± SD. The p-values for interaction, treatment (P vs. NK), and time (baseline, 30 min before immersion, and 39 min after completion of water immersion) were obtained using the repeated two-way analysis of variance, followed by Bonferroni's multiple comparison tests, if necessary.

## 4 Discussion

Previous evidence has suggested that oral intake of NK may enhance peripheral blood flow in humans [15]. The objective of the present study was to evaluate the effects of acute NK intake on skin temperature in healthy men during and after cold water immersion of the hands. The main finding of this study was that the skin temperatures of the middle finger, palm, and back of the right hand were significantly higher after cold water immersion following NK intake than placebo intake. Meanwhile, no significant differences were observed between NK and placebo intake across other parameters, such as CO and related parameters, LF/HF ratio, and blood pressure. The results of this study indicate that a single acute dose of oral NK accelerated the recovery of peripheral skin temperature after cold water immersion, but did not have any impact on whole-body haemodynamic response or sympathetic nervous activity in healthy adult men.

In this study, the skin temperature of the middle finger, palm, wrist, and back of the right hand, and the mean skin temperature of the whole body decreased during cold water immersion of both hands in a 10 °C-water bath for 1 min. Moreover, the skin temperature of the middle finger, palm, and back of the right hands was significantly higher during the recovery period after NK supplementation than after the placebo. To the best of our knowledge, this is the first study to demonstrate that a single dose of oral NK enhances recovery from cold-induced decrease in peripheral skin temperature in humans. A previous study observed a significant decline in plasma kininogen levels after injection of 5,000 FU of NK-like protease into the duodenum in rats, indicating an increase in plasma kinin, a degradation product of kininogen. Kinin functions as a vasodilator through the synthesis of nitric oxide and prostacyclin [22]. In the current study, we did not evaluate peripheral skin blood flow and plasma levels of kininogen and kinin; however, it might be possible that elevation of plasma kinin levels through the decline of plasma kininogen after NK intake enhanced peripheral skin blood flow. A study conducted by Pan et al. [22] also reported a significant elevation of plasma angiotensinogen levels, a precursor of angiotensin I and angiotensin II, after an NK-like protease injection, suggesting the possibility of an inhibition of angiotensin I production from angiotensinogen or a reduction of renin. Although we did not determine the activity of angiotensin converting enzyme (ACE), it is suggested that the inhibition of ACE after NK intake may be conducive to enhanced peripheral blood flow through vasodilation, due to the reduction in angiotensin II production and the preservation of kinin [11,23,24]. Further studies are needed to clarify the precise mechanism by which NK intake enhances recovery from cold-induced decrease in peripheral skin temperature. The results of this study imply the potential applicability of NK. In peripheral vasoconstrictive coldness, which is more common in women, peripheral blood vessels contract excessively with cold stimulus, thus, resulting in reduction of peripheral blood flow, which leads to decline of skin temperature [25]. Thermoregulation with cutaneous blood flow (i.e., reflex cutaneous vasoconstriction and vasodilation) are both impaired with aging [26]. NK might have potential applications in many cases, such as in women who have peripheral vasoconstrictive coldness and in the elderly.

In the current study, HR and LF/HF ratio, as indicators of sympathetic nervous activity, were significantly increased due to cold water immersion, whereas no change in other parameters was found. Moreover, no treatment interaction was observed in any of the haemodynamic-related parameters. Reduction of peripheral skin temperature from cold exposure occurs due to sympathetically mediated vasoconstriction, including upregulation of α2-adrenergic receptor activity [27]. There are varying experimental protocols with water temperatures ranging from 0 °C to 28 °C [28,29], demonstrating that skin temperature drastically drops below 14 °C, with an effective increase in muscle sympathetic nervous activity, arterial blood pressure, and HR [28]. A lower temperature immersion for a longer duration creates cold-induced vasodilation (CIVD) [30,31]. Thus, in this study, we used a relatively higher water temperature of 10 °C and a shorter duration of 1 min to reduce pain and intolerance, and to prevent CIVD. Accordingly, in this study, HR and LF/HF ratios were significantly increased due to cold water immersion of both hands after both NK and placebo supplementation, with no significant difference. This is probably because a single dose of oral NK with the current immersion protocol would not have impacted peripheral circulation to the point of inhibition of the baroreflex and whole body haemodynamic and sympathetic nervous activity in healthy men.

In the current study, resting systolic and diastolic blood pressures in the sitting position were not significantly different at 90 min and 160 min after NK intake compared with placebo intake. This result is consistent with our previous findings that a single dose of 2,000 FU of NK did not affect the resting systolic and diastolic blood pressures of healthy normotensive men [8]. Meanwhile, another study by Kim et al. [11] reported a reduction in both systolic and diastolic blood pressures among participants whose baseline systolic blood pressure was 130–159 mmHg after a daily intake of 2,000 FU of oral NK for 8 weeks, but not after 4 weeks. These results indicate that to observe a reduction in blood pressure at a safe dose, oral NK would need to be taken for longer than 4 weeks, and that this effect might be more pronounced for borderline or mild hypertensive individuals than normotensive individuals. Additional studies are warranted to systematically explore the effect of NK intake on blood pressure.

This study has some limitations. First, all participants were healthy men, and healthy women were not included. Our reasons for enrolling only male participants are because women have significantly lower skin temperature, which is associated with a higher percentage of body fat [32], and because healthy women are more sensitive to cold stimuli than healthy men [33]. In general, both sex, age [34], body fat [35], and smoking [36] affect the dynamics of skin temperature. Therefore, to reduce confounding, we recruited only healthy men participants with a relatively narrow age range, normal body fat percentage, who were non-smokers. However, this means changes in skin temperature induced by cold water immersion among individuals who are more sensitive to cold, such as women, the elderly, and smokers, were not investigated in this study. Again, women are more sensitive to cold stimuli [33], and recovery of finger skin temperature and skin blood flow after water immersion has been shown to be significantly slower in women than men [37]. Reduced peripheral skin blood flow commonly causes not only sensitivity to cold, but also clinical conditions such as neck tension, headaches, pressure ulcers, and ischemic necrosis. Recruiting participants who are more sensitive to cold, such as women and elderly, would provide further evidence of the efficacy of oral NK intake. Second, we did not directly measure skin blood flow. However, resting peripheral skin temperature is mainly regulated by skin blood flow [38]. In another study, a single dose of 2,000 FU of NK, the same as that in the current study, enhanced skin blood flow of the middle fingers of healthy participants [15]. Therefore, we assumed that the elevation of peripheral skin temperature after cold water immersion following oral NK intake could be attributed to the enhancement of peripheral skin blood flow. Additionally, the skin blood flow and vasodilation/vasoconstriction substrates in the blood were not determined. Additional studies are needed to address these limitations.

## 5 Conclusion

This study supported our hypothesis that a single dose of oral NK accelerates the recovery of peripheral skin temperature after cold water immersion. In contrast, the acute administration of 2,000 FU of NK did not affect changes in HR, CO, sympathetic nervous activity, and blood pressure compared with a placebo in healthy men. Although the underlying mechanisms by which NK intake may influence peripheral skin temperature remain unclear, the results of the current study highlight the potential implications of NK for the prevention of excessive vasoconstriction; for example, in individuals with cold sensitivity (i.e. women), elderly individuals, and associated medical conditions.

## Ethics statement

All experimental procedures and measurements conformed to the tenets of the Declaration of Helsinki and were approved by the Institutional Review Board of Tokyo Medical University (approval no. #T2019-0047). This trial was registered with the University Hospital Medical Information Network (approval no. UMIN000045964).

## Production notes

### Author contribution statement

Noriko Nara and Yuko Kurosawa: Conceived and designed the experiments, Performed the experiments, Analysed and interpreted the data, Wrote the paper.

Sayuri Fuse-Hamaoka, Miyuki Kuroiwa, Tasuki Endo and Riki Tanaka: Performed the experiments, Analysed and interpreted the data.

Ryotaro Kime: Conceived and designed the experiments, Performed the experiments.

Takafumi Hamaoka: Conceived and designed the experiments, Performed the experiments, Wrote the paper.

### Data availability statement

Data will be made available on request.

## Declaration of competing interest

The authors declare the following financial interests/personal relationships which may be considered as potential competing interests: This work was supported by Japan Bio Science Laboratory Co., Ltd, Osaka, Japan, who also provided the nattokinase supplements and placebo. The funder had no role in the design of the study; in the collection, analyses, or interpretation of data; in the writing of the manuscript, or in the decision to publish this work.
